# An overview of lumbar anatomy with an emphasis on unilateral biportal endoscopic techniques: A review

**DOI:** 10.1097/MD.0000000000031809

**Published:** 2022-12-02

**Authors:** Qiang Zhang, Yongan Wei, Li Wen, Chang Tan, Xinzhi Li, Bo Li

**Affiliations:** a Department of Orthopedics, China Three Gorges University, Renhe Hospital, Yi Chang, China.

**Keywords:** lumbar spine, spinal nerve, unilateral biportal endoscopy

## Abstract

Unilateral biportal endoscopy (UBE) is a major surgical technique used to treat degenerative lumbar diseases. The UBE technique has the advantages of flexible operation, high efficiency, and a large observation and operation space. However, as a typical representative of minimally invasive techniques, UBE still needs to complete a wide range of decompression and tissue resection in a narrow working space, resulting in many surgery-associated injuries. Therefore, it is necessary to reduce complications by familiarity with the anatomy of the lumbar spine. Based on the UBE technique, this review article provides historical and current information on the anatomical structures of the lumbar vertebrae, such as the articular process, pedicle, lamina, ligamentum flavum, nerve root, intervertebral disc, and artery supply.

## 1. Introduction

Lumbar degenerative diseases are among the most common clinical diseases, including lumbar disc herniation, lumbar spinal stenosis, lumbar instability, and lumbar spondylolisthesis.^[1]^ Lumbago and leg pain and/or nerve damage caused by these diseases are widespread and complicated in the clinic, seriously affecting patients’ health and quality of life.^[2]^ According to statistics, the time-point prevalence of low back pain in the general population is 18.3% and 30.8% during 1 month, which is the first reason workers retire early.^[3]^

Generally, lumbar degenerative diseases are treated conservatively, and surgical intervention is often required when the effect of non-surgical treatment is poor or when symptoms of severe nerve damage occur. In the past decades, mainstream spinal surgery has been open surgery. However, in recent years, the rise of spinal endoscopy techniques has opened a new direction for minimally invasive spinal surgery. Its development direction is mainly small incision or pipeline technique. Contrary to the development trend of endoscopic techniques in general and thoracic surgeries, the single-channel endoscopic technique was the first to become popular in spinal endoscopy with a light source, observation lens, and surgical instruments passing through the same pipeline. Although the current single-channel endoscopic spinal technique has been widely used in surgical removal of the protruding nucleus pulposus, surgical decompression of spinal stenosis, and even intervertebral fusion,^[4]^ it is still a targeted technique with relatively limited surgical field and scope, and relatively high technical requirements for surgeons.

Unilateral biportal endoscopy (UBE) has recently become popular. Compared with the traditional open technique, unilateral 2-channel endoscopy is still a “point exposure” technique, with a high incidence of surgery-associated injury, including the dural sac, outlet nerve root, and dural injury on the walking nerve root.^[5]^ Park^[6]^ observed 643 patients who underwent UBE surgery and found that the incidence of dural injury was 4.5%. Lee^[7]^ reported that the incidence of dural injuries was as high as 13.2%. Therefore, surgeons need to be familiar with the anatomy of the lumbar spine. In this study, the anatomical structure of the lumbar spine involved in unilateral 2-channel endoscopy was reviewed comprehensively.

## 2. Overview of lumbar anatomy

### 2.1. The articular process and the pedicle

Compared to the cervical and thoracic vertebrae, the facet of the lumbar vertebrae is closer to the sagittal plane, and the facet angle to the sagittal plane increases gradually from the top down, averaging 33.^[8]^ The facet of the lumbar spine has less mobility in the rotation direction but more mobility in the direction of flexion and extension. Studies^[9]^ have shown that the smaller the angle between the lumbar spine and sagittal plane, the more likely it is that degenerative lumbar spondylolisthesis will occur. It is worth noting that the superior articular process can be used as one of the anatomical markers to determine the position of the disc. Studies^[10]^ have shown that the distance between the highest point of the superior articular process and the central plane of the disc is 1 to 2 mm. Therefore, the position of the disc can be inferred from the position of the highest point of the superior articular process. Pedicle shape was related to race, sex, weight, and other factors. Morita K^[11]^ measured the morphological parameters of the pedicle in 227 patients on computed tomography (CT). From L1 to L5, the angle of the pedicle to the sagittal plane gradually increased by approximately 10 to 30°, the width of the pedicle gradually increased by approximately 7 to 16 mm, and the height of the pedicle decreased by approximately 12 to 16 mm. The spinal nerve extends around the lower edge of the pedicle of the same ordinal vertebrae and exits the spinal canal.

### 2.2. Spinal nerve

It is essential to master the anatomical structure of the nerve tissue in the lumbar canal, because dural injury is the most common complication of UBE. The nerve tissue in the lumbar canal consists of the dural sac and 6 pairs of spinal nerves (L1-L5, S1) that emanate from the dural sac. After the spinal nerve emanates from the dural sac, it travels diagonally, laterally, ventrally, and caudally and leaves the spinal canal around the lower edge of the pedicle at the same ordinal level (approx. 1 mm). The spinal nerve was uplifted from the dural sac, and the width of L1 to L5 was approximately 15 mm, and that of S1 was approximately 10 mm. The angle between the spinal nerve and dural sac on the coronal plane gradually decreased from top to bottom. L1 was 40° to 45°, L2 and L3 were generally between 30° and 40°, L4 and L5 were generally between 25° and 30°, and S1 was generally 15° to 20°.^[12]^

The spinal nerves are composed of anterior and posterior roots. The dorsal root has a distensible nodule composed of centripetal sensory fibers, which are responsible for receiving nerve impulses from bodily receptors and are closely associated with spinal radicular pain. The location of the DRG varies significantly among different spinal nerve segments. Most dorsal root ganglia from L1 to L3 are located in the intervertebral foramen. Most of the dorsal root ganglia of L4 (approx. 85%) are located in the foramina, a few are located in the spinal canal (approx. 10%), and a few are located outside the foramina (approx. 5%). Most of the dorsal root ganglia of L5 (approx. 70%) are located in the foramina, some (approx. 20%) are located in the spinal canal, and a few (approx. 10%) are located outside the foramina. The dorsal root ganglion of the S1 spinal nerve is mostly located in the spinal canal (approx. 75%), and partially in the foramina (approx. 25%). This partly explains why high central and paracentral disc herniations often do not cause clinical symptoms, whereas extreme lateral disc herniations often cause severe root symptoms. Foraminal stenosis in the L5/S1 segment was less likely to cause root symptoms.^[13]^

Spinal nerves L1 to L5 divide into anterior and posterior branches after leaving the foramina, whereas S1 usually does not branch. According to the number of dorsal root ganglions and anterior roots, spinal nerves can be divided into 3 types^[14]^: type A, 1 dorsal root ganglion and 1 anterior root; type B, 1 dorsal root ganglion and 2 anterior roots; and type C, 2 dorsal root ganglia and 2 anterior roots. Approximately 65% of L4 nerve roots were type B and 30% were type C. More than 80% of L5 roots were type B and approximately 15% were type C.

### 2.3. The relationship between the intervertebral disc and spinal nerves

Spinal nerves L1 to L4 originate below the corresponding disc. The distance from the L1 to L3 spinal nerve outlet to the corresponding disc distance was generally 15 mm, and that from the L4 spinal nerve was generally 10 mm; 62.5% of L5 spinal nerves were generated below the corresponding disc, 12.5% were generated above the corresponding disc, and 25% were generated at the disc level. 75% of S1 spinal nerves originate above the corresponding disc and 25% originate at the disc level, which explains why most disc herniations in L5/S1 are of the axillary type.^[15]^

The Kambin triangle, a safe area for posterolateral approach discectomy and interbody fusion, consists of the lateral edge of the dural sac, outlet nerve root, and upper edge of the lower vertebral body. The upper edge of the lower vertebra is the bottom edge of the Kambin triangle, which consists of the distance between the outer edge of the dural sac and the inner edge of the outlet nerve root. The vertical edge of Kambin triangle is the distance from the outlet nerve root of the dural sac to the upper edge of the lower vertebral body. In the L1/2 to L3/4 planes, the base of the Kambin triangle is approximately 11 to 13 mm, and L4/5 to L5/S1 is approximately 14 to 17 mm.^[16]^

### 2.4. The relationship between the lamina and the disc

At the rear, the upper and lower boundaries of the intervertebral space are at the lower edge of the upper lamina, the upper edge of the lower lamina, and the upper edge of the lower lamina does not exceed the upper edge of the vertebral body. In L1 to L5, the distance between the upper edge of the lower lamina and upper edge of the vertebral body was approximately 10 to 11 mm, and in S1, the distance between the upper edge of the lower lamina and upper edge of the vertebral body was approximately 14 mm. This is because the intervertebral space is usually partially covered by the lower edge of the upper lamina, which exceeds the lower edge of the vertebral body by approximately 2 to 4 mm. The width of the left-right intervertebral space gradually increased from top to bottom, and the width of the intervertebral space was 16 to 19 mm from L1/2 to L3/4. The lamina space widths of L4/5 was approximately 23 mm, and that of L5/S1 was over 30 mm, respectively.^[17]^

### 2.5. The arterial supply

The lumbar artery emerges from the celiac artery and runs laterally around the vertebral body, giving off the major psoas branches about the middle of the vertebral body (anteroposterior diameter). Before entering the foramina, the lumbar plexus branches emerged from the lumbar artery to the spinal nerve junction, nourishing the lumbar plexus. Vertebral, lateral, and dorsal branches are emitted at the outer orifice of the foramina. A branch of the vertebral body enters the spinal canal below the pedicle and nourishes the posterior wall of the vertebral body. When the disc is exposed sideways, it can damage the arteries and cause bleeding. The dorsal branches bypass the spinal nerves and nourish the posterior paraspinal muscles. In addition, the dorsal branch sends out spinal nerve branches and nourishes the spinal nerve. The lateral ramus passes caudally to the transverse process and terminates at the abdominal wall.^[18]^

### 2.6. The ligamentum flavum

A critical point of the UBE technique is how to quickly and safely break through the ligamentum flavum into the spinal canal. The ligamentum flavum was divided into shallow and deep layers. The superficial ligamentum flavum has a light-yellow fibrous structure with a 2.5 to 3.5 mm thickness. It is connected to the base of the spinous process and ventral side of the lower edge of the cephalic lamina at the proximal end, to the dorsal side of the caudal lamina at the distal end, and fused with the interspinous ligament at the midline. The deep ligamentum flavum is relatively thin (approx. 1 mm) and is connected to the ventral lamina proximally and distally. The attachment point of the deep ligamentum flavum varies from one segment to another. The lower the segment, the greater is the lamina covered by the ligamentum flavum. The ligamentum flavum covers approximately 50% of the cephalic lamina at L1 to L2 and 70% at L4 to L5. At the caudal lamina, the ligamentum flavum covered 1 to 2 mm at L1 to L2, and 5 to 6 mm at L5 to S1.^[19]^ Laterally, the deep ligamentum flavum extended into the foramina, forming the posterior wall of the foramina.^[20]^

### 2.7. The application of unilateral 2-channel endoscopic techniques

With the increasing popularity of spinal endoscopy and the expansion of surgical indications, single-channel spinal endoscopy has shown significant limitations in lateral recess decompression, contralateral decompression, and severe spinal stenosis decompression, and its surgical efficiency is relatively low owing to the limitations of the supporting surgical tools.^[21]^ Because to these limitations, the UBE technique has been widely used in recent years. UBE is a surgical technique in which 2 small percutaneous incisions in the posterior ipsilateral spine are used to establish an observation and working channel for internal and external spinal canal surgery. As early as 1996, Kambin^[22]^ used bilateral dual-channel endoscopy and the UBE technique for the first time to remove the nucleus pulposus. De Antoni et al further improved the UBE technique in the same year.^[23]^ However, due to the lack of attention paid to spinal endoscopy by the spine community at that time, and the shunt effect of single-channel foraminoscopy,^[24]^ which was introduced in 1999, the UBE technique did not attract much attention at that time.

In recent years, with the rise of the endoscopic technique, scholars have realized that owing to the dual-channel design of UBE, the observation and working channels are relatively independent, with the advantages of convenient and flexible operation, ample observation and operation space, the use of conventional surgical instruments, and high efficiency.^[21]^ In addition, to remove the nucleus pulposus, the UBE technique is also suitable for extensive decompression of spinal stenosis and interbody fusion. Kim et al analyzed 60 patients who underwent discectomy using the UBE technique and found that the clinical effect of patients in the UBE group was similar to that of traditional open surgery. However, the hospital stay was relatively short, and early symptoms of low back pain were relatively mild.^[25]^ Heo^[26]^ compared the application of the UBE technique, traditional microendoscopy technique, and single-channel spinal endoscopy technique for simple decompression in patients with central spinal stenosis and found that the clinical effect of the UBE technique is similar to that of other technologies; however, compared with traditional techniques, it has the advantages of a lighter degree of early postoperative low back pain and relatively high comfort. Kang^[27]^ compared UBE lumbar fusion with microendoscopic-assisted lumbar fusion and found that the 2 had similar clinical results, with relatively small amounts of blood loss and postoperative drainage.

## 3. Conclusion and outlook

ssion and tissue resection in a narrow working space, resulting in many surgery-associated injuries. Therefore, familiarity with the anatomy of the lumbar spine is necessary to reduce complications.

## Author contributions

**Conceptualization**: Yongan Wei.

**Formal analysis**: Bo Li.

**Methodology**: Li Wen.

**Resources**: Chang Tan.

**Writing – original draft**: Qiang Zhang.
